# A comprehensive review of influenza B virus, its biological and clinical aspects

**DOI:** 10.3389/fmicb.2024.1467029

**Published:** 2024-09-04

**Authors:** Muhammad Awais Ashraf, Muhammad Asif Raza, Muhammad Nabeel Amjad, Ghayyas ud Din, Lihuan Yue, Bei Shen, Lingdie Chen, Wei Dong, Huiting Xu, Yihong Hu

**Affiliations:** ^1^CAS Key Laboratory of Molecular Virology and Immunology, Institutional Center for Shared Technologies and Facilities, Pathogen Discovery and Big Data Platform, Shanghai Institute of Immunity and Infection, Chinese Academy of Sciences, Shanghai, China; ^2^University of Chinese Academy of Sciences, Beijing, China; ^3^Pediatric Department, Nanxiang Branch of Ruijin Hospital, Shanghai, China

**Keywords:** influenza B virus, isolation, identification, immunity and antiviral strategies, public health

## Abstract

Influenza B virus (IBV) stands as a paradox, often overshadowed by its more notorious counterpart, influenza A virus (IAV). Yet, it remains a captivating and elusive subject of scientific inquiry. Influenza B is important because it causes seasonal flu outbreaks that can lead to severe respiratory illnesses, including bronchitis, pneumonia, and exacerbations of chronic conditions like asthma. Limitations in the influenza B virus’s epidemiological, immunological, and etiological evolution must be addressed promptly. This comprehensive review covers evolutionary epidemiology and pathogenesis, host-virus interactions, viral isolation and propagation, advanced molecular detection assays, vaccine composition and no animal reservoir for influenza B virus. Complex viral etiology begins with intranasal transmission of influenza B virus with the release of a segmented RNA genome that attacks host cell machinery for transcription and translation within the nucleus and the release of viral progeny. Influenza B virus prevalence in domesticated and wild canines, sea mammals, and birds is frequent, yet there is no zoonosis. The periodic circulation of influenza B virus indicates a 1–3-year cycle for monophyletic strain replacement within the Victoria strain due to frequent antigenic drift in the HA near the receptor-binding site (RBS), while the antigenic stability of Yamagata viruses portrays a more conservative evolutionary pattern. Additionally, this article outlines contemporary antiviral strategies, including pharmacological interventions and vaccination efforts. This article serves as a resource for researchers, healthcare professionals, and anyone interested in the mysterious nature of the influenza B virus. It provides valuable insights and knowledge essential for comprehending and effectively countering this viral foe, which continues to pose a significant public health threat.

## 1 Introduction

**Figure 1:**
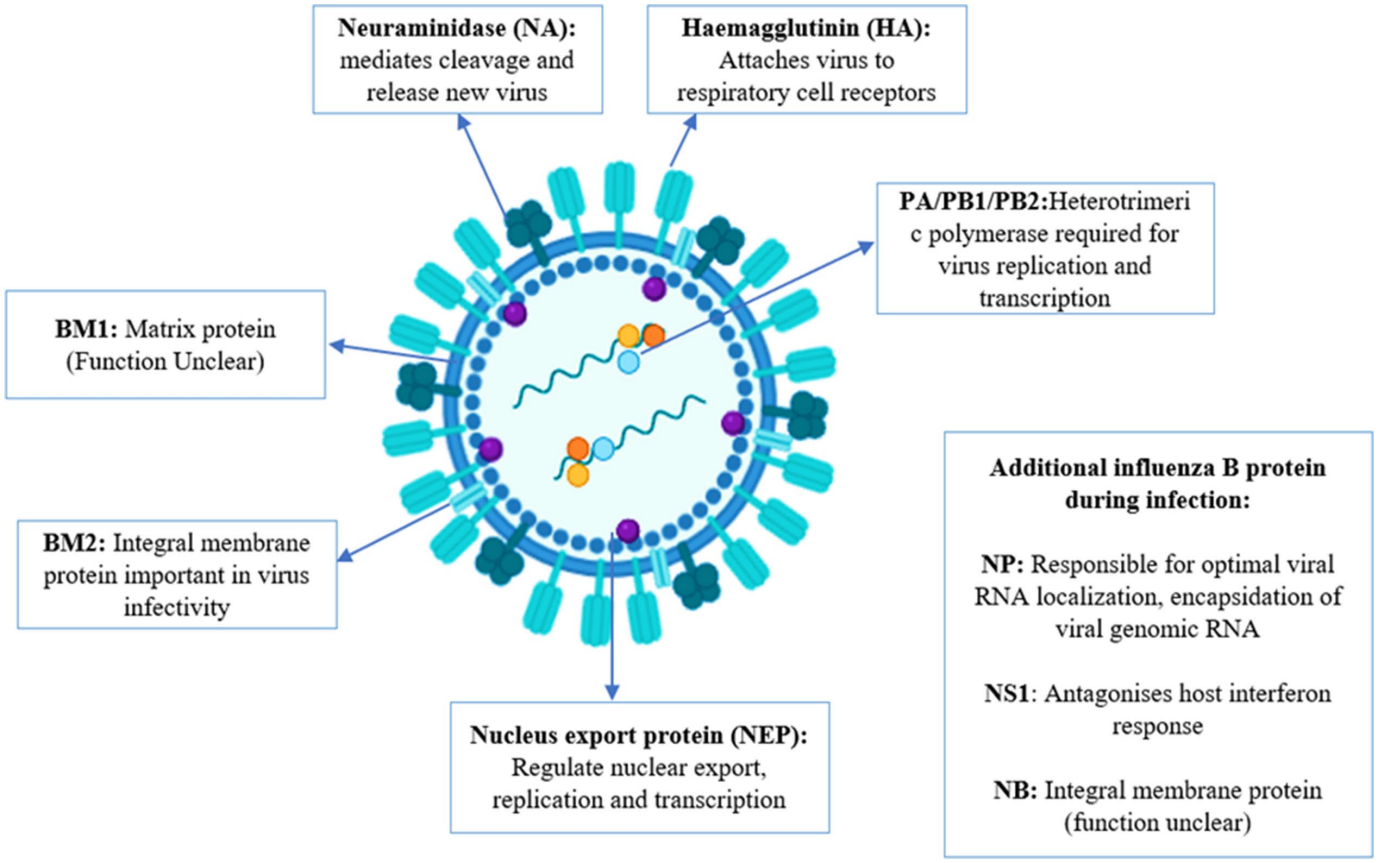
Overview of influenza B genome and brief details of proteins.

The Spanish flu emerged in 1918 and was identified as the influenza A H1N1 virus. In 1940, the first outbreak of respiratory illness occurred in children, with clinical signs similar to those of the influenza A virus (IAV). This lead to the identification of a new virus, named the influenza B virus (Francis Jr, 1940). It is now understood that influenza A and B viruses co-circulate among the human population across different age groups with varying degrees of severity. Unlike the influenza A virus, the influenza B virus has no established animal reservoir and poses no pandemic risk (Jackson et al., 2011). The influenza B virus can pose significant challenges during annual epidemics, yet it often receives less attention compared to other strains, while influenza C and D are not as prevalent. According to the WHO and CDC, influenza causes approximately 650,000 deaths each year, with the influenza B virus contributing to 20 to 30% of these fatalities. The purpose of this review is to provide a comprehensive analysis of influenza B, focusing on its epidemiology, genetic evolution, and impact on public health. Understanding the significance of influenza B is crucial due to its substantial role in seasonal flu epidemics, particularly affecting vulnerable populations such as children and the elderly, and the ongoing challenges it presents in vaccine development after the low prevalence of B/Yamagata and public health management (Figure 1).

The influenza B virus has segmented, negative-sense, single-stranded RNA composed of 8 segments. It includes three surface glycoproteins (HA, NA, and NB), a BM2 ion channel, a nuclear export protein (sometimes referred to as BNS2 or NEP), a matrix protein (BM1), a nonstructural protein (NS1), and a nucleoprotein (NP), which encodes three polymerase proteins (PB1, PB2, and PA). The virus belongs to the Orthomyxoviridae family (Palese, 2007). The influenza A virus is classified into various subtypes based on the combination of hemagglutinin (HA) and neuraminidase (NA) surface proteins, leading to substantial antigenic variation and the potential for pandemics. Influenza A has 18 HA subtypes (H1 to H18) and 11 NA subtypes (N1 to N11). The most significant influenza subtypes are H1N1 and H3N2, which contribute to various epidemics and pandemics (Shao et al., 2017). Influenza B virus does not have accessory proteins PA-X or PB1-F2 while influenza A has these proteins (Krumbholz et al., 2011; Shi et al., 2012). The proteins of influenza A and B show differences in composition, length, and functions (Jackson et al., 2011). Influenza A virus has various subtypes while influenza B viruses have been divided into two antigenically and genetically different lineages: B/Yamagata/16/1988-like and B/Victoria/2/1987-like virus (Rota et al., 1990; McCullers et al., 2004; Vijaykrishna et al., 2015).

## 2 Genetic evolution and phylodynamics of IBV

**Figure 2:**
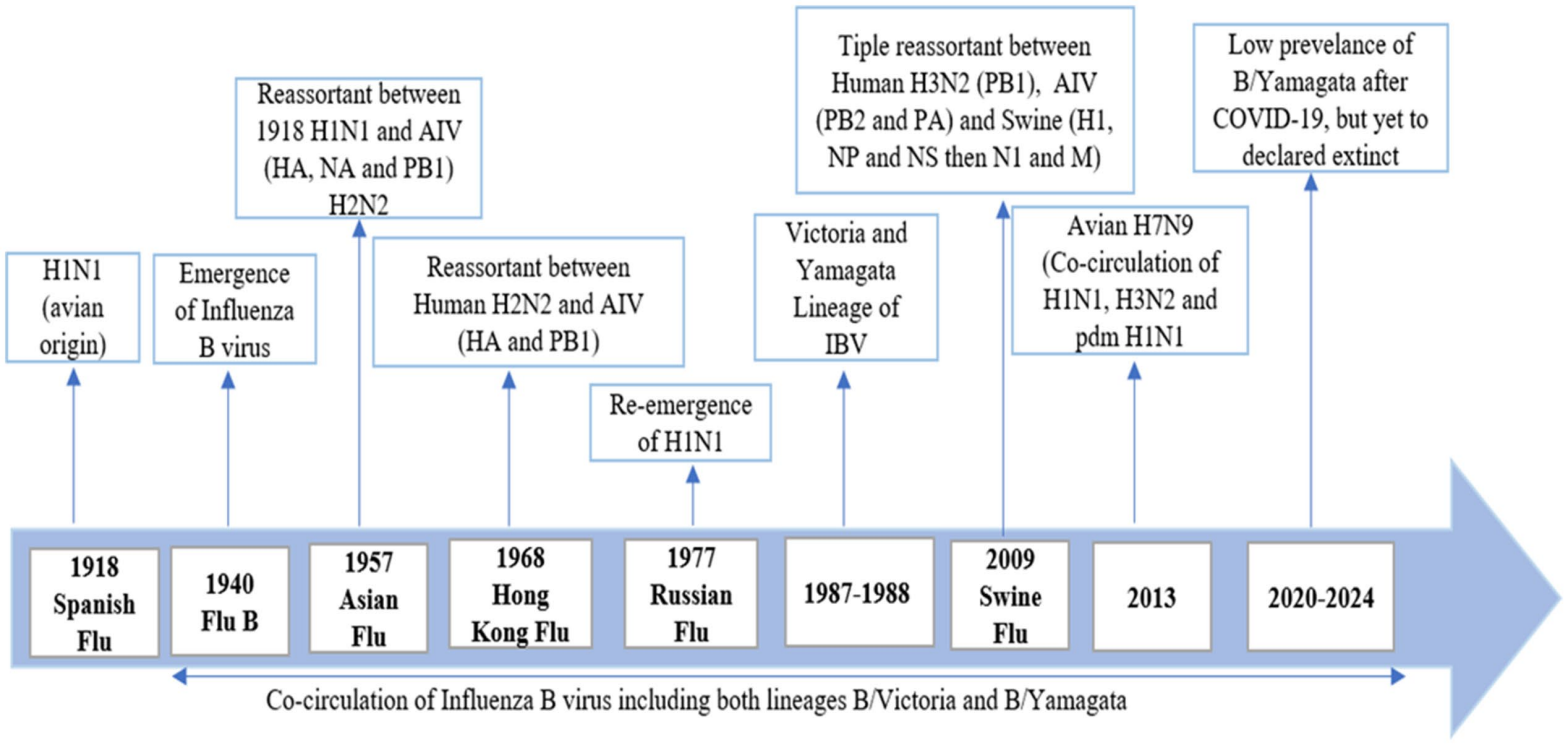
History and evolution of influenza virus.

Influenza viruses are a significant cause of morbidity and mortality worldwide, posing major public health challenges. Among these, two types of influenza viruses circulate widely in human populations: influenza A and influenza B. Influenza A viruses, particularly the A (H3N2) subtype, are known for their widespread impact (McCullers and Hayden, 2012), are generally associated with higher rates of hospitalization and mortality compared to influenza B, the latter still significantly contributes to the global influenza disease burden, causing approximately one-third of global influenza cases each year and severe disease, especially in children (Yang et al., 2012). Influenza B viruses are classified into two distinct lineages: B/Yamagata/16/88 (Yamagata lineage) and B/Victoria/2/87 (Victoria lineage) (Kanegae et al., 1990). These lineages emerged in the 1970s and have co-circulated globally since at least 1983, with both lineages alternating in regional dominance (Kanegae et al., 1990). The differentiation is marked by HA features, and by 1988, 27 amino acid (a.a.) differences were noted in the HA1 domains of these lineages (Rota et al., 1990). Minimal a.a. changes were observed in the HA2 domains at that time, and phylogenetic analysis indicated separate lineages for the HA2 domains of both B/Yamagata/16/88-like and B/Victoria/2/87-like viruses (Rota et al., 1992). Genome-wide analyses suggest that reassortment plays a crucial role in the evolution of influenza B viruses, with frequent exchanges observed between the Yamagata and Victoria lineages across all eight gene segments (Chen and Holmes, 2008). Despite the slower pace of antigenic change in influenza B compared to influenza A, the Victoria lineage experiences more rapid lineage turnover, exhibiting distinct epidemiological patterns such as longer persistence in local regions before wider dissemination. Victoria-lineage viruses tend to show faster antigenic drift rates, averaging 3.9 to 5.1 years, whereas the Yamagata lineage has an average antigenic drift rate of 6.3 to 7.2 years (Langat et al., 2017). Nonetheless, Yamagata viruses tend to infect an older population and exhibit an epidemiological pattern of alternating antigenic dominance between seasons (Figure 2).

The evolutionary patterns of these lineages reveal that Yamagata-lineage viruses consist of two coexisting clades: clade 2 (B/Massachusetts/02/2012) and clade 3 (B/Wisconsin/1/2010) (Skowronski et al., 2014). In contrast, Victoria-lineage viruses have diversified into two dominant monophyletic clades (clades 1A and 1B) since 2011 (Langat et al., 2017). The PB1 and PB2 phylogenetic trees display considerable divergence akin to the HA gene, with the PB1, PB2, and HA genes consistently originating from a singular lineage (Dudas et al., 2015). Historically, influenza B virus epidemics have been sporadic compared to influenza A, but recent years have seen significant changes, with substantial epidemics reported globally, such as the high activity observed in Europe in 2015 (Barr et al., 2016). Since 2001, both influenza B lineages have been co-circulating worldwide during each epidemic season. They can coexist in different proportions within the same season and in specific regions (Chen and Holmes, 2008). Most studies suggest that the B/Victoria lineage is more prevalent than the B/Yamagata lineage in younger populations (Yang et al., 2018). A comprehensive analysis conducted using the Global Influenza B Surveillance (GIBS) database has highlighted significant differences in the age demographics affected by B/Victoria and B/Yamagata influenza infections. The findings reveal that B/Yamagata infections predominantly affect older age groups compared to B/Victoria. In most countries studied, B/Victoria infections are primarily found in younger individuals, typically between 0 to 25 years old, with a notable peak among children under 10 years of age. On the other hand, B/Yamagata infections often exhibit a bimodal age distribution pattern. This means there are two distinct peaks in infection rates: one among children under 10 years old and another, albeit smaller, among adults aged 25 to 50 years (Yang et al., 2018).

The introduction of quadrivalent vaccines, which include strains from both influenza B lineages, has been recommended to mitigate issues often seen with trivalent vaccines that cover only one lineage. This limitation frequently results in reduced vaccine effectiveness (Tisa et al., 2016). Efforts to develop a universal vaccine capable of targeting both B lineages are a critical public health priority. Recent research indicates that both lineages have undergone selective sweeps preceding resurgences. Victoria-lineage viruses have shown more pronounced changes in the HA gene, including nucleotide deletions, and have co-evolved with neuraminidase (NA) and internal gene segments. In contrast, Yamagata-lineage viruses exhibit stronger seasonal fluctuations due to antigenic drift in the NA protein. While antibody cross-protection between the two B lineages is generally considered low, some studies suggest significant levels of cross-reactive serum antibodies (Skowronski et al., 2011). Experimental evidence indicates that vaccination with the B/Yamagata lineage can induce cross-antibody responses to the Victoria lineage, though the reverse is less effective. Studies involving B-cell memory and monoclonal antibodies (mAbs) derived from individuals vaccinated with the quadrivalent seasonal vaccine (IIV-4) have confirmed the immunological dominance of B/Yamagata HA (Liu et al., 2019).

## 3 Epidemiology of IBV

The 1889–1890 Russian flu emerged in 1889 and lasted for four years in 4 waves. The Russian flu was caused by *Myxovirus* and lately confirmed as influenza H2N2 (Berche, 2022a). It caused 257 million deaths in 1890, 574,000 in 1891 and 534,000 in 1892 (Honigsbaum, 2011). The 1918–1919 Spanish flu was caused by influenza A virus H1N1. It caused 50–100 million deaths (Berche, 2022b). The 1957–1958 Asian flu was caused by an H2N2 strains of influenza A virus. While influenza B co-circulated during this period but their role was minor as compared to influenza A (MacKellar, 2007). The 1968–1969 Hong Kong flu caused by H3N2 strain of influenza A virus. Influenza B did not play a significant role in this period but co-circulate in this season (Tang et al., 2008). The 1977 Russian flu was caused by the reemergence by a novel H1N1 strain of influenza A virus similar to the 1918 virus. Influenza B did not contribute to this epidemic (Kalyar et al., 2024). The 2009–2011 Swine flu mainly caused by H1N1 (Lim and Mahmood, 2011). The cases of IBV infection can vary rapidly during the influenza season (Toback et al., 2012). In a period between 1985 and 2000, a single influenza B virus lineage, B/Yamagata in the 1990s and B/Victoria in the late 1980s, dominated the season of influenza, but after 2001, both lineages have been spreading in the southern and northern hemispheres (Bodewes et al., 2011; Sauerbrei et al., 2014; Paul Glezen et al., 2013; Kim et al., 1979). In 20th century, influenza B virus caused 23.4% cases in each flu season. In the Netherlands, the B/Yamagata lineage has spread every influenza season between the periods of 1999 and 2007, except for the season of 2002–2003. The B/Victoria lineage spread from 2001 to 2006, except in 2004 (Thompson et al., 2004). Early thoughts suggested that IBV causes a less severe disease than IAV. However, the rates of mortality, morbidity, and hospitalization (except during the IAV pandemic) did not support this evidence. (Thompson et al., 2004). IBV is normally thought to be less severe as compared to IAV/H3N1 but more severe than IAV/H1N1 in the last ten years (Thompson et al., 2004; Thompson et al., 2010; McCullers and Hayden, 2012; Claas et al., 1995; Grant et al., 2009; Kawai et al., 2011). IBV causes disease in a population of all age groups, and the disease is spreading more among young adults and children. B/Yamagata lineage causes more infection in the young age group as compared to B/Victoria (McCullers and Hayden, 2012). According to the CDC report, IBV accounted for 34% of influenza deaths during the period between 2004–2005 and 2009–2010, except for the pandemic of 2009 (Thompson et al., 2010). From the Netherlands data, a 29% ratio of IBV was detected in respiratory samples from the period of 1992–1993 to 2006–2007 (Dijkstra et al., 2009). In the Netherlands, an analysis performed with serum samples taken from 720 children showed that 72% of the children at the age of 7 years had developed antibodies against IBV, which is indicative of a high attack rate (Bodewes et al., 2011). On the other side, in Germany, a seroprevalence study conducted from 2008 to 2010 indicated that 47% of individuals under the age of 17 developed specific IgG antibodies against IBV (Sauerbrei et al., 2014). Data from 12 pediatric hospitals in Canada between 2004 and 2013 indicated that the mortality rate among hospitalized patients (aged ≤16 years) with influenza B (1,510 cases) was higher compared to those with influenza A (2,645 cases), with 16 deaths (1.1%) for influenza B and 10 deaths (0.4%) for influenza A (Tran et al., 2016). This study also revealed that otherwise healthy children (aged ≥10 years) infected with influenza B had a significantly higher likelihood of requiring intensive care unit (ICU) admission than those with influenza A, with an odds ratio of 5.79 (95% confidence interval 1.91–17.57). Additionally, a systematic review of 126 studies emphasized the severity of influenza B infections in children, showing that influenza B accounted for 22–44% of all influenza-related pediatric deaths from 2004 to 2011, excluding the 2009–2010 period (Paul Glezen et al., 2013). More recent data from the US (2010–2016) supports these findings, indicating that influenza B viruses were responsible for 16–52% of all influenza-associated pediatric deaths (Shang et al., 2018). Fatal cases of influenza B in children are often linked to bacterial pneumonia and cardiac injury, with the latter being a major cause of influenza B-related deaths in this age group (Paddock et al., 2012). In comparison, almost all the children at the age of 7 developed antibodies against at least one IAV strain (Toback et al., 2012; Sauerbrei et al., 2014). The seroprevalence is based on many factors, including the spreading of the influenza virus (A/H1N1, B, and A/H3N2), the endurance of the evident antibody feedback, and a geographic map showing the need for more comprehensive studies. Based on CDC data, an average of 24% of respiratory samples with infection collected in the United States from 2001–2002 to 2010–2011 (2009 is excluded because it was the pandemic year) tested positive for IBV through reverse transcriptase polymerase chain reaction (RT-PCR) (Toback et al., 2012). Surveillance data from Europe showed that an average of 23% of influenza-infected respiratory samples tested positive for IBV simultaneously (Dijkstra et al., 2009). Influenza B caused 20% of the influenza cases in each season from 2000 to 2018 in 21th century. Influenza B type contribute 20% in 118 seasons, 20 to 50% in 115 seasons and above 50% in 45 seasons. Influenza B usually peaked in August to September in the countries of the southern hemisphere 1.1 month later than influenza A. Average proportion of influenza B cases that were reported in a season caused by B/Victoria lineage. In 27 seasons out of 84, 20% co-circulation of the both the lineages were reported. While B/Victoria contributed 70% of the cases in a season while B/Yamagata reported 30% of the cases. Influenza B/Victoria infected age between 10 and 13 while B/Yamagata infected older people. The proportion of B/lineage vaccine mismatch was 42.9% in countries of the northern hemisphere and 54.2% I the countries of the southern hemisphere (Caini et al., 2019).

After 2020, B/Yamagata circulation has not been confirmed (Caini et al., 2023). In 2021, India reported 3 B/Yamagata detections, while Bulgaria, Afghanistan, Nigeria, Mexico, and the United States reported 1 B/Yamagata detection. In 2022, North Korea and Germany reported 1 B/Yamagata detection. In 2023, Cuba reported 1 B/Yamagata detection. There were many new cases reported in the early months of 2024, but none of the sequences were uploaded to GISAID. No B/Yamagata detection has been confirmed since 2021. There is a myth that influenza B/Yamagata extinct but it is too early to declare the extinction of B/Yamagata. The absence of B/Yamagata sequences post-March 2020 highlights the importance of continued molecular surveillance to monitor lineage dynamics and detect potential resurgence events. Overall, the combined analysis of epidemiological and molecular surveillance data provides valuable information on the temporal trends and dynamics of influenza activity, informing strategies for influenza prevention, control, and preparedness. According to WHO Flu Net and GISAID, Table 1 represents a summary of influenza cases in the last 5 years (GISAID, n.d.; WHO, 2024).

**Table 1:** Reported cases of influenza virus during the last 5 years (till June 30, 2024).

Year	Total influenza	IAV	%	IBV	%	IBV Victoria%	IBV Yamagata %
2019	789,011	657,369	83.3	131,642	16.7	92.9	7.1
2020	471,106	303,038	64.3	168,068	35.7	98.6	1.4
2021	116,225	82,181	70	30,044	29.3	99.9	0.1
2022	363,251	329,688	90.8	33,563	9.2	100	0
2023	892,260	712,497	79.85	179,763	20.14	100	0
2024	792,610	577,090	72.80	215,520	27.19	100	0

Sources: Flu Net.

## 4 Pathogenesis and replication cycle of IBV

**Figure 3:**
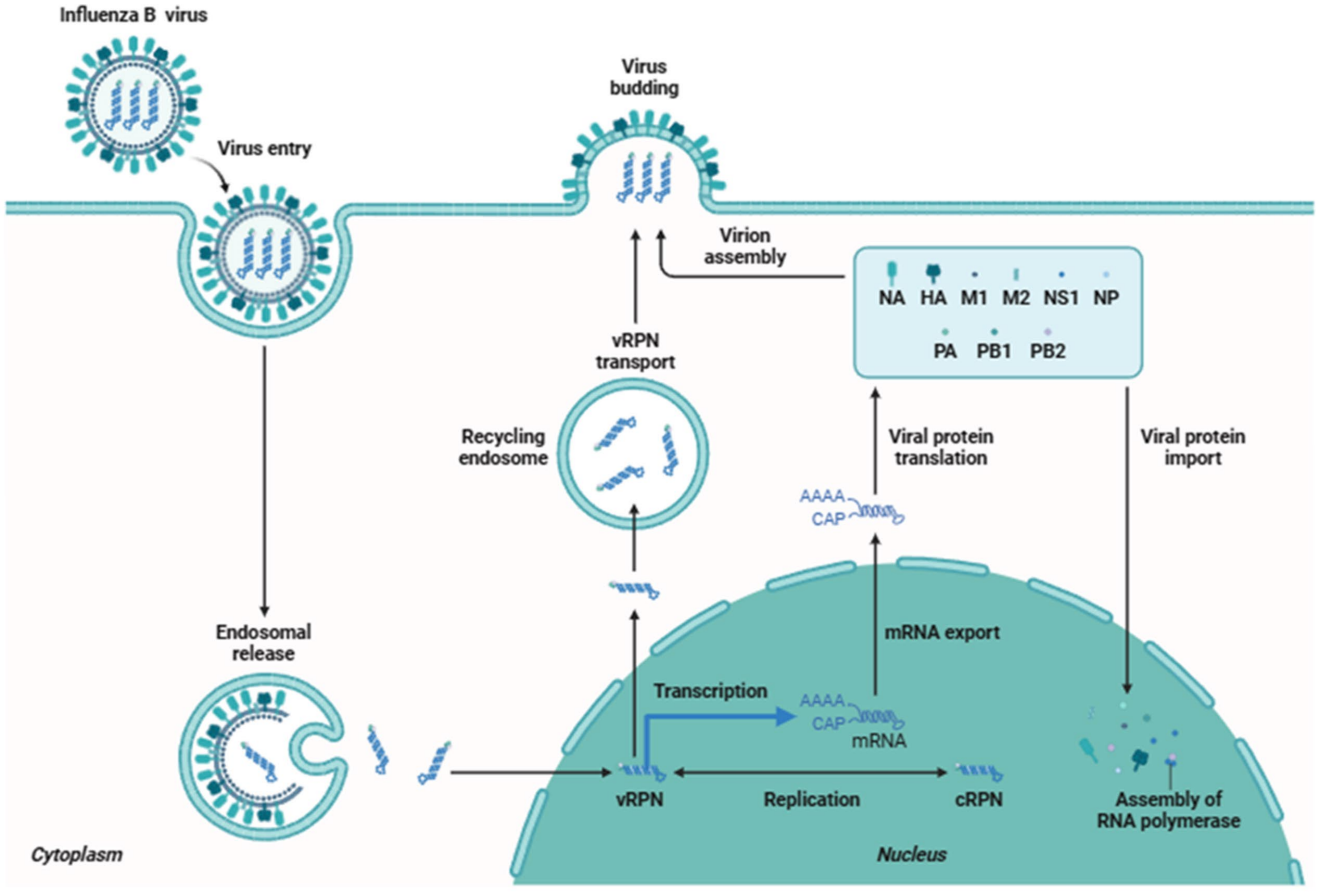
Replication cycle of influenza B virus.

A sore throat, coughing, nasal discharge, and fever are signs of an IBV infection (Wright and Kawaoka, 2007). Overall, despite possible differences in consequences, IBV and IAV caused illnesses are identical (Irving et al., 2012). Neurological and muscular symptoms (Moon et al., 2013; Thabet et al., 2013), cardiologic issues (Frank et al., 2010; Paddock et al., 2012; Taremi et al., 2013), and subsequent bacterial infections (Paddock et al., 2012; Taremi et al., 2013; Aebi et al., 2010; Scaber et al., 2011) are also possible signs of IBV. The period between the commencement of the illness and death in fatal cases of influenza was shown to be shorter for IBV than for the 1918, 1957, 1968, and 2009 pandemics of IAV (Paddock et al., 2012). The influenza B virus is more prevalent in children, but it also infects older people and adults. The Victoria lineage of the influenza virus is more common in children than the Yamagata lineage. The respiratory route serves as an entry point for the influenza B virus into the body, predominantly through inhaling respiratory droplets that carry the virus. These droplets are produced during activities such as coughing, sneezing, or talking by an infected individual (Kutter et al., 2018). Infected individuals can spread the from 1 day before symptoms appear and up to 7 days after becoming sick. The virus attaches to the respiratory epithelial cells lining the airways, primarily in the nose and throat. The viral surface protein called hemagglutinin binds to specific receptors on the surface of the host cells. The virus then enters the cells using receptor-mediated endocytosis. Once inside the host cell, the virus releases its genetic material, which is in the form of eight single-stranded RNA segments (Matsuoka et al., 2013; Kapoor et al., 2014). The viral RNA serves as a template for the synthesis of new viral proteins and the replication of the viral genome. This process occurs in the host cell’s nucleus. The newly synthesized viral proteins and RNA segments are assembled to form new virus particles. These viral particles then exit the host cell by budding from the cell membrane or causing the cell to burst (lysis) (Chauhan and Gordon, 2022). The released virus can infect neighboring cells, leading to the spread of infection throughout the respiratory tract. The infection triggers an immune response in the body. The immune system recognizes the presence of the virus and initiates an inflammatory response to control the infection. Immune cells, such as macrophages and natural killer cells, target and eliminate infected cells. The adaptive immune response, involving B cells and T cells, produces antibodies that can neutralize the virus and help clear the infection (Figure 3).

The replication cycle begins when the virus accesses the columnar epithelial cells of the respiratory tract, overcoming the protective mucus layer by using its neuraminidase (NA) glycoprotein to cleave mucin and sialic acid. The hemagglutinin (HA) glycoprotein then binds the virus to the exposed epithelial cells, facilitating endocytosis via clathrin and caveolin-dependent mechanisms. Within the acidic environment of the lysosome, the viral matrix protein (M1) dissociates from the ribonucleoprotein (RNP) complex, releasing viral RNA that is subsequently imported into the host cell nucleus for replication (Mohsan Ullah Goraya and Munir, 2015).

## 5 No animal reservoir for IBV

Influenza A virus circulates among humans and various other wild animals, including dogs, pigs, horses, and chickens. Nevertheless, IBV has been isolated from diverse species such as grey seals (*Halichoerus grypus*), harbor seals (*Phoca vitulina*), and dogs (*Canis lupus familiaris*), understanding the virus’s ability to infect these animals (Chang et al., 1976; Leyva-Grado et al., 2012). Moreover, serological evidence confirms IBV infection in specific animal species residing in close proximity to humans. These species include guinea pigs (*Cavia porcellus*), dogs (*Canis lupus familiaris*), pigs (*Sus domesticus*), ruminants (*Bornean orangutans*) (*Pongo pygmaeus*), horses (*Equus ferus caballus*), western lowland gorillas, western common chimpanzees (*Pan troglodytes verus*), and zoo birds (Horimoto et al., 2014; Brown et al., 1995; Kawano et al., 1978; Buitendijk et al., 2014; Romvary et al., 1980; Ran et al., 2015; Ohishi et al., 2002; Bodewes et al., 2013). Distinct antibodies specific to IBV have been identified in various species of wild pinnipeds, such as grey seals (*Halichoerus grypus*), Caspian seals (*Phoca caspica*), South American fur seals (*Arctocephalus australis*), and harbor seals (*Phoca vitulina*). However, these antibodies have not been detected in harbor porpoises (*Phocoena phocoena*) (Bodewes et al., 2014; Blanc et al., 2009). Although IBV demonstrates the ability to infect certain wild species, there is no evidence of transmission to humans. Influenza B virus isolated from seals in Netherland but no evidence of transmission from seals to human observed. Influenza B virus continue to infect seals and support the notion that seals could be reservoir for influenza B virus (Bodewes et al., 2013). The primary specific reason influenza B virus does not have an extensive animal reservoir is its host specificity. Unlike influenza A virus, which has the ability to infect a wide range of species due to its diverse subtypes and high genetic variability, influenza B virus is primarily adapted to humans and, to a lesser extent, seals. It has been shown that influenza B virus also susceptible to pigs. A serological study indicated that influenza B antibodies detected in 7.3% of tested swine herds (Ran et al., 2015). Influenza B virus also detected from the respiratory tract of ferrets and pigs (Pica et al., 2012; Elderfield et al., 2015). There are certain reason influenza B does not have animal reservoir. Firstly, influenza B viruses have evolved to bind specifically to receptors found in the human respiratory tract. This receptor binding specificity limits their ability to infect and replicate in other species, as different animals may have different types of receptors that influenza B cannot efficiently bind to. Secondly, Influenza B virus has less genetic diversity compared to influenza A. This limits its ability to adapt to and evolve in different animal hosts. The genetic makeup of influenza B is fine-tuned for human hosts, reducing its ability to spill over into other species. Finally, influenza A viruses can undergo antigenic shift, where different subtypes reassort their gene segments, potentially creating new viruses that can infect new host species. Influenza B does not have this capability, which limits its ability to jump to and establish itself in new animal hosts. Influenza B host range is currently not well understood or may likely expand over time (Jang and Seong, 2020).

## 6 Experimental animal models for IBV

The study of the pathogenicity of influenza B virus infection indicates the importance of employing experimental model animals to analyze vaccine protective efficiency and immunogenicity in the context of human disease. Numerous studies involving IBV have been conducted on various animal models, including ferrets (*Mustela putorius furo*) and mice (*Mus musculus*). Additionally, pigs, guinea pigs, dogs, Syrian golden hamsters (*Mesocricetus auratus*), cynomolgus macaques (*Macaca fascicularis*), and cotton rats (*Sigmodon hispidus*) have been practically inoculated with various IBV strains, as detailed in Table 2 (Ran et al., 2015; Huang et al., 2014; Ottolini et al., 2005; Song et al., 2015; Rarey et al., 1987; Kim et al., 2009; Maassab et al., 1982; Pica et al., 2012; Hirst, 1947; Pica et al., 2011; Davis et al., 1990; Davis, 1987; Takatsy and Romvary, 1969; Reeve and Gerendas, 1981; Kitano et al., 2010). Virus multiplication has been observed in all laboratory animals except for dogs, and a specific experimental study in pigs demonstrated pig-to-pig circulation infected with B/Victoria strains (Ran et al., 2015). Clinical signs were widely observed, and the severity of the disease was contingent on the IBV strains employed for inoculation (Huang et al., 2014; Pica et al., 2012; Davis, 1987). In the 1980s and 1990s, a mouse-adapted human IBV strain (B/Lee/40) was introduced to experimental mice to examine IBV’s characteristics in the progression of Reye’s syndrome in children (Davis, 1987). Through intravenous inoculation, mice exhibited numerous pathological, clinical, and virological features identical to those observed in children with Reye’s syndrome. The titration of IBV was also scrutinized in ferrets (Deshmukh, 1985; Rarey et al., 1984; Mukhopadhyay et al., 1992; Rarey, 1985).

**Table 2:** Experiment animal detail for IBV.

Animal species	Inoculation route	Virus replication	Clinically sign	References
Cotton rats (*Sigmodon hispidus*)	IN	Yes (URT/LRT)	No data	Ottolini et al. (2005)
Mice (*Mus musculus*)	IN/IV	Yes	Mortality	Maassab et al. (1982), Hirst (1947), Pica et al. (2011), Davis et al. (1990), and Davis (1987)
Syrian golden hamsters (*Mesocricetus auratus*)	IN	Yes (URT/LRT)	Absent	Reeve and Gerendas (1981)
Ferrets (*Mustela putorius furo*)	IN	Yes (URT/LRT)	Pneumonia detected by histopathology, hearing losses, Strain dependent, including weight loss, nasal discharge, fever,	Huang et al. (2014), Velkov (2013), and Wang et al. (2012)
Guinea pigs (*Cavia porcellus*)	IN	Yes (LRT/URT)	Strain-dependent histological changes in LRT and URT	Pica et al. (2012)
Pigs (*Sus domesticus*)	IN/nasopharyngeal cavity/IT	Yes, and transmission	Absent to influenza-like symptoms, including pneumonia	Ran et al. (2015) and Takatsy and Romvary (1969)
Dogs (*Canis lupus familiaris*)	IN	No	Absent	Reeve and Gerendas (1981)
Cynomolgus macaques (*Macaca fascicularis*)	Conjunctiva/IN/IT	Yes (URT/LRT)	Histopathology revealed a decreased appetite, fever, and pneumonia, and weight loss	Kitano et al. (2010)

IN, intranasally; LRT, lower respiratory tract; IV, intravenously; URT, upper respiratory tract.

## 7 Isolation of IBV

### 7.1 Egg inoculation

For the amplification of the influenza virus, embryonic eggs constitute a reliable supply (Goodpasture et al., 1919). The most typical source of eggs is chicken; however, eggs from ducks and other species can also support viral replication. The fact that mammalian influenza viruses primarily connect to 2,6-linked sialic acid while avian influenza viruses preferentially bind to 2,3-linked sialic acid is an important factor to take into account. The cells in the allantoic cavity primarily express 2,3-linked sialic acid, while the cells in the amniotic cavity express both 2,3- and 2,6-linked sialic acid, and this distinction partly determines the inoculation technique (Ito et al., 1997). As a result, mammalian influenza viruses will reproduce better in the amniotic cavity than avian influenza viruses do in the allantoic cavity. However, injecting into the allantoic cavity is simpler, and most influenza viruses can adapt to this method, making it the preferred approach necessary for vaccine production. An overview of the standard procedure for embryonated egg inoculation is provided in the sections that follow. The “Manual for the Laboratory Diagnosis and Virological Surveillance of Influenza” is available to the public on the WHO website and provides a comprehensive explanation of the egg inoculation technique (WHO, 2011). Isolating the IBV from embryonated eggs is a commonly used technique in virology. This method capitalizes on the virus’s ability to replicate in embryonated eggs, facilitating its easy isolation and propagation (Table 3).

**Table 3:** Comparison of identification techniques.

Method	Advantages	Disadvantages	Potential for surveillance	References
Conventional RT-PCR/PCR	Relatively specific and sensitive; allow sequence analysis	Not ideal for high-throughput not easily quantitative;	Time-consuming for large screens	Ferreira et al. (2009)
Real-time PCR	High specificity and sensitivity, Cost-saving	Special equipment needed, extensive optimization	Reliable and sensitive method for clinical laboratories	Templeton et al. (2004)
Multiplex PCR	comparatively sensitive and specific; capable of testing several targets in a single experiment; available for downstream sequence analysis; time and money-saving	Multiple primers can cause non-specific amplification	The most common method used for surveillance detection for identifying subtypes using different segments especially combined with real-time PCR	Templeton et al. (2004)
Microarrays	Specific, sensitive, high throughput, and large number of targets in a single assay	Downstream analysis complex, extensive equipment needed	Good for surveillance in core facilities or high laboratory	Rogers et al. (2019)
Pyrosequencing	Sensitive and accurate, high throughput	Sequence length limitation, time-consuming	Very useful for the detection of molecular markers of drug resistance	Lau et al. (2019)
NASBA	Relative specific, sensitive, and quantitative	Require optimization for primer selection	It is a suitable option for field applications because it does not require expensive devices, especially when the surveillance targets are well-identified	Moore et al. (2010)
LAMP	Fast, simple, cost-saving	Require extensive optimization for primer design to achieve high sensitivity	It is the perfect detection technique for remote locations and impoverished nations because it requires a standard laboratory water heat or bath block for the reaction	Mahony et al. (2013)

NASBA, nucleic acid sequencing-based amplification; LAMP, loop-mediated isothermal amplification.

### 7.2 Cell culture

Cell cultures are a practical substitute for embryonated eggs for the amplification of many primary influenza virus isolates, making them useful for the production of virus stocks. Cells are readily available in large quantities when needed and can be frozen for long-term storage. In terms of repeatability and virus amplification effectiveness, cell lines offer a good alternative to embryonated eggs, given the unpredictability of the isolation rates in these cells. Additionally, cell culture technologies have the potential for quicker vaccine production in the case of a pandemic, as well as a less expensive and more adaptable setup during vaccine research. The use of continuous tissue culture cell lines in the creation of vaccines is strictly regulated by the relevant health organizations due to their propensity to cause tumors. In continuous cultures of Madin Darby Canine Kidney (MDCK) epithelial cells, the majority of viral isolates and vaccine candidates are now multiplied effectively (Tobita et al., 1975). These cells were first described in 1966 (Gaush et al., 1966) and are obtained from the kidney of an adult female cocker spaniel. It has been demonstrated that numerous IAV and IBV strains may replicate in MDCK cells. Additionally, to quantify samples containing influenza viruses, a plaque assay technique was developed in MDCK cells (Gaush and Smith, 1968). Trypsin significantly increases the growth of IAV and IBV on MDCK cells, making it possible to detect influenza viruses from human specimens with high sensitivity (Tobita et al., 1975; Meguro et al., 1979; Davies et al., 1978). By cleaving the HA0 precursor of the viral surface glycoprotein into the HA1 and HA2 subunits, trypsin increases the infectiousness of the influenza virus (Lazarowitz and Choppin, 1975; Klenk et al., 1975). The fusion of the viral membrane with the endosomal membrane, which releases the genetic material of the virus into the newly infected cell, requires the cleavage of the influenza virus HA (Helseth et al., 1991). Exogenous trypsin is not necessary for all influenza viruses, although some lab strains and the extremely deadly H5 and H7 strains are among them. Due to their resistance to trypsin-containing media and the fact that their cell surface proteins include both 2,3- and 2,6-linked sialic acids, MDCK cells are perfect for the propagation of both mammalian and avian influenza viruses (Ito et al., 1997).

## 8 Identification of IBV

### 8.1 Reverse transcriptase PCR (RT-PCR)

RT-PCR is a vital method for detecting and analyzing RNA viruses, including influenza B virus. It involves sample collection, RNA extraction, reverse transcription, PCR amplification, and PCR cycling. The RNA is converted into cDNA, amplified, and detected using methods like gel electrophoresis or real-time PCR. This method helps track strain prevalence, antiviral drug resistance development, and contribute to influenza outbreak surveillance and control. In one of the studies, 105 influenza A and B samples, including 10 embryonated chicken egg-isolated viruses, 751 cell culture isolates, and 344 primary clinical specimens, were collected during the period from 2005 to 2015. These virus samples were collected from Australia, United States of America, and another region within the Global Influenza Surveillance and Response System (WHO GISRS). The RNA was extracted from the specimen using an extraction kit (Table 4). Then the virus is confirmed by real-time RT-PCR. In this study, all the samples collected for virus types B/Yamagata and B/Victoria were 100% positive, including 6/6 B/Yamagata and 46/46 B/Victoria. In primary specimens, 14 out of 17 samples were positive for B/Yamagata, and 10 out of 11 samples were positive for B/Victoria. (Zhou et al., 2017).

**Table 4:** Overview of FDA approved influenza vaccines (Grohskopf et al., 2024).

Vaccine type	Manufacturing method	Approved age range	Special feature
Cell-based flu shot (Flucelvax Quadrivalent)	Virus grown in cell culture	6 months and older	Destroyed viruses, there is a chance of adverse events like allergy
Standard-dose flu shots (e.g., Afluria, Fluarix, FluLaval, Fluzone)	Virus grown in eggs	6 months and older	History of severe allergic reaction
Recombinant flu shot (Flublok Quadrivalent)	Made using recombinant technology	18 years and older	Egg-free; includes three times the antigen to enhance the ability of the immune system to respond
Adjuvanted flu shot (Fluad Quadrivalent)	Virus grown in eggs with an adjuvant	65 years and older	Contains an adjuvant for a stronger immune response
High-dose flu shot (Fluzone High-Dose Quadrivalent)	Virus grown in eggs	65 years and older	Contains four times the antigen for a stronger immune response
Live attenuated flu nasal spray (FluMist Quadrivalent)	Attenuated live flu viruses	2–49 years	Administered through the nasal spray; not recommended for certain groups. (severe medical conditions and pregnancy)

### 8.2 Real-time PCR

Real-time PCR, also known as quantitative PCR (qPCR), is a molecular biology technique used to detect and quantify specific RNA or DNA sequences, including the influenza B virus. It uses fluorescent dyes or probes to monitor amplification, producing fluorescence directly proportional to the PCR product. Clinical samples are collected, followed by RNA extraction, reverse transcription, and real-time PCR setup. The cDNA containing the virus is subjected to amplification, with fluorescence intensity monitored in real-time. Between 2010 and 2013, clinical samples, including pharyngeal and nasal swabs, from 169 patients exhibiting influenza-like symptoms were collected. Among these samples, 20 out of 149 were identified as positive for the influenza B virus. For B/Yamagata and B/Victoria, RT-PCR assays were performed. Out of 20 positive samples, 6 samples were detected as positive for the B/Yamagata lineage, and 14 samples were detected as positive for the B/Victoria lineage. The highly specific and sensitive B/Yamagata and B/Victoria RT-PCR assays will help discriminate the influenza B virus lineage in clinical samples (Nakauchi et al., 2014).

### 8.3 Multiplex PCR

Multiplex PCR is a method enabling simultaneous amplification of multiple DNA or RNA sequences, ideal for detecting diverse influenza B virus strains. It employs specific primers targeting various viral genome regions, and involves sample collection, RNA extraction, and reverse transcription. These steps prepare cDNA for PCR, where multiple primer sets amplify distinct genome segments in one reaction. Optimized PCR conditions ensure efficient amplification. Post-amplification, products are analyzed via gel electrophoresis or real-time PCR, offering insights into virus prevalence, genetic diversity, and vaccine efficacy, vital for epidemiology and clinical diagnostics. Some clinical samples of influenza A and B were isolated from medical centers, schools, factories, and hospitals during the outbreak in Shenzhen from 1994 to 2006. The Center for Disease and Prevention of Foshan, Shenzhen, and Wuhan Ministry of Health China provided 189 throat swabs from patients with different respiratory diseases. For 189 clinical samples, conventional culture techniques and multiplex PCR were performed. Influenza A (subtype N1) detected by multiplex PCR was 67, and for influenza B, it was 14, totaling 42.9% (81 samples). However, influenza A was detected by conventional culturing at 35 and influenza B at 11, totaling 24.3% (46 samples). 81 samples were identified by multiplex RT-PCR and agarose gel electrophoresis analysis. However, only 46 samples were identified as influenza A and B by conventional methods. These results indicated that multiplex PCR is more sensitive, reliable, and provides more accurate results (Wu et al., 2008).

### 8.4 Microarray

A microassay for influenza B virus is a laboratory technique that enables rapid and sensitive detection of the virus in a small sample volume. One common approach is the microarray-based assay, which involves immobilizing specific probes on a solid surface, such as a glass slide or microchip, to capture viral nucleic acids from a clinical samples (Schena et al., 1995; Pease et al., 1994). The process involves sample collection, nucleic acid extraction, labeling of target nucleic acids, hybridization, scanning, and data analysis. Microarray-based assays offer several advantages, including high throughput, rapid detection, sensitivity and specificity, and a small sample volume. These methods are crucial for influenza surveillance, vaccine development, and understanding the epidemiology of the virus. However, the specific technology and protocols used for microassays can vary depending on the laboratory and available equipment. Overall, microarray-based assays play a crucial role in influenza surveillance, vaccine development, and understanding the epidemiology of the virus. All the human parainfluenza samples were provided by the Colorado Department of Health and Prevention. These samples were collected for the detection of influenza A and B viruses by using the microarray technique. A total of 85 samples were tested on the B-chip, including parainfluenza type 1 and two influenza A subtypes (H3N2 and H1N1) as the negative control and 62 different positive samples of influenza B that originated worldwide during the years 1946–2005. Out of 65 samples, two samples of IBV were not identified by the microarray. When these two samples were treated with gel, they showed no signal, meaning multiplex RT-PCR amplification failed to detect all three gene segments. When the microarray was repeated, these two samples showed a signal. The negative control and influenza A were correctly identified as negative. Overall, for the detection of influenza B virus, the B-chip results show a specificity of 100% and sensitivity of 97%. (Dankbar et al., 2007).

### 8.5 Pyrosequencing

Pyrosequencing is a DNA sequencing method that determines the nucleotide sequence of a DNA fragment. It is a sequencing-by-synthesis technique that measures the release of pyrophosphate (PPi) during DNA synthesis (Kim et al., 2009). It is applied to study organisms, including viruses like influenza B, to understand their genetic diversity and evolution (Maassab et al., 1982). Pyrosequencing involves sample collection, RNA extraction, reverse transcription, PCR amplification, the pyrosequencing reaction, the luciferase reaction, and data analysis. It offers several advantages for studying the influenza B virus and its genetic variations, including high throughput, accuracy, sensitivity, and rapid results. However, it requires specialized equipment and expertise, and its application in the laboratory may vary depending on available resources and protocols. Pyrosequencing is valuable for studying viral evolution, drug resistance, and immune escape, but it requires specialized equipment and expertise. To check if the pyrosequencing could be used for clinical samples with similar specificity and sensitivity, 105 influenza B-positive samples were received for testing from late 2016 to June 2017. Out of 105 samples, there were 16 B/Victoria, 82 B/Yamagata, and 7 B/Victoria-Deletion. This assay detected the first B/Victoria 2 deletion variant in Australia, and no B/Victoria deletions were detected afterward. These same samples were used to perform RT-PCR to compare the results with the results of pyrosequencing. The seven B/Victoria-Deletion samples were also detected as B/Victoria lineages. Pyrosequencing can detect the B/Victoria deletion variant virus and is found to be more informative and sensitive (Pica et al., 2012).

### 8.6 Nucleic acid sequence based amplification (NASBA)

NASBA is a powerful isothermal nucleic acid amplification technique (Pasick, 2008) used to detect and quantify specific RNA targets, particularly for RNA viruses like influenza B. It operates at a constant temperature, typically 41–42°C, making it suitable for point-of-care and resource-limited settings. NASBA involves sample collection and RNA extraction, primer design, amplification monitoring, detection, and quantification. The reaction involves annealing primers to the target RNA and reverse transcription of the RNA into complementary DNA (cDNA). Fluorescence data collected during the NASBA reaction can be used to determine the presence and quantity of the influenza B virus in the original sample. NASBA is used to test various strains of human and animal viruses. Analytical sensitivity analysis indicates a threshold of 10 copies in direct amplification. Eighty-nine throat/nasal swabs were collected from hospitalized children (0–16 years old) at Herriot Hospital, Lyon, France, to evaluate this assay. Among 89 swabs, real-time NASBA identified 10 samples positive for influenza and 2 samples positive for influenza B virus. This highly sensitive method enables the simultaneous detection of influenza A and B in a single reaction, completed within 3 h (Van Aarle et al., 2006).

### 8.7 Loop-mediated isothermal amplification (LAMP)

Loop-mediated isothermal amplification (LAMP) is a powerful nucleic acid amplification technique used for the rapid and sensitive detection of specific RNA or DNA targets, including the influenza B virus (Notomi et al., 2000). LAMP operates at a constant temperature and is known for its simplicity, speed, and suitability for point-of-care testing. It involves sample collection and RNA extraction, LAMP primer design, LAMP amplification, visual detection, and confirmation. LAMP offers several advantages for the detection of influenza B virus and other RNA viruses, including rapid and isothermal amplification, high sensitivity and specificity, and point-of-care application. LAMP is widely used in research, clinical diagnostics, and field applications, showing promise as a reliable and cost-effective tool for early detection and monitoring of viral infections. Some clinical samples were tested for LAMP. There was an epidemic of influenza A and B in Japan in 2004/2005. Eighty-three nasopharyngeal swabs were collected from patients aged 67 years. During the real time LAMP (RT-LAMP) procedure, 71 samples (83.5%) were detected as positive. Thus, RT-LAMP was thought to be a reliable, rapid, and sensitive method. In addition, detection and subtyping of influenza can also be performed by RT-LAMP (Ito et al., 2006).

## 9 Host immune response against IBV

**Figure 4:**
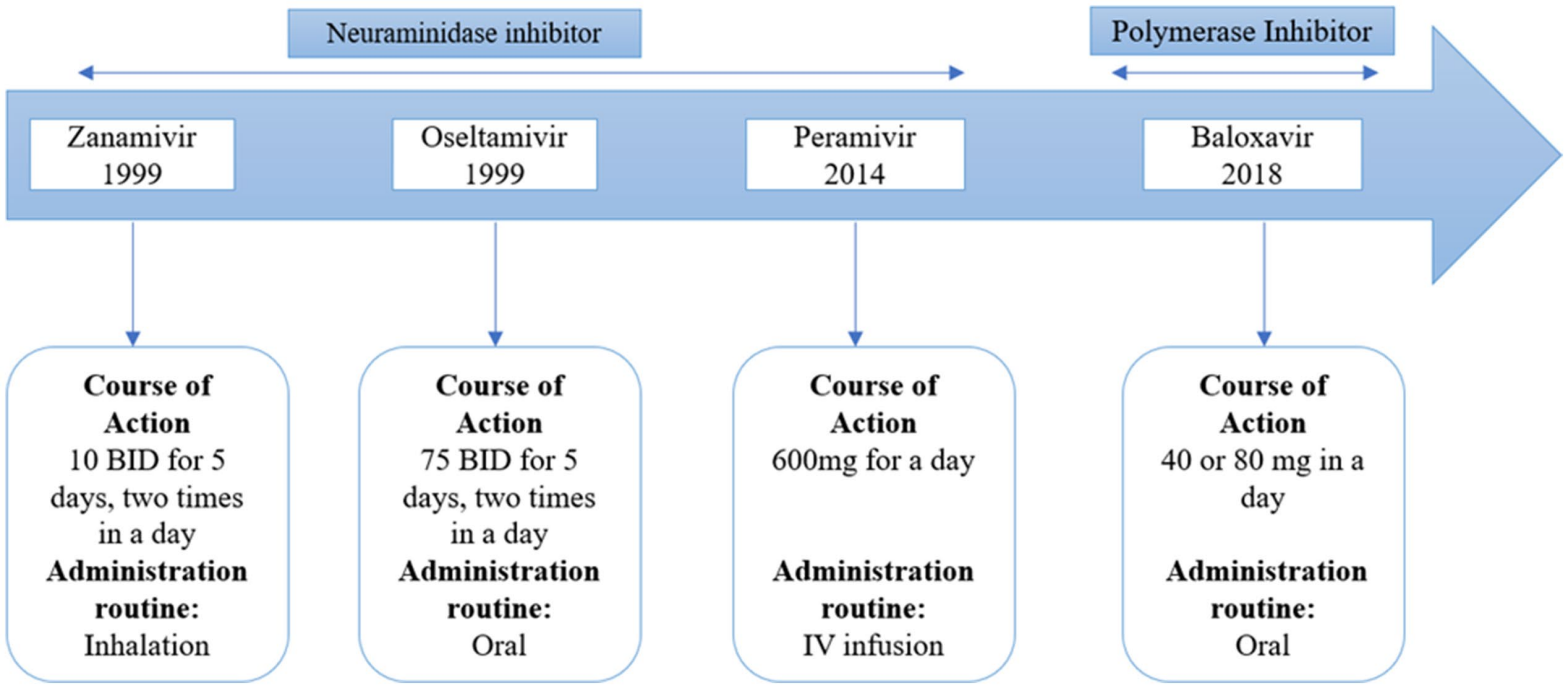
Globally approved antivirals drugs against influenza B virus.

Influenza B viruses are a significant cause of respiratory illness, particularly in children. The host immune response to the influenza B virus is critical for controlling the infection and preventing severe disease. This discussion aims to cover the immunology of influenza B viruses, encompassing the innate and adaptive immune responses, along with the current understanding of protection mechanisms and immune evasion (Figure 4).

### 9.1 Innate immune response

The initial immune response acts as the primary defense mechanism against influenza B viruses. Pattern recognition receptors (PRRs), including Toll-like receptors (TLRs), retinoic acid-inducible gene-I (RIG-I), and melanoma differentiation-associated protein 5 (MDA5), detect the virus. This recognition triggers the generation of type I interferon (IFNs) and pro-inflammatory cytokines, activating immune cells and initiating the adaptive immune response (Kawai, 2009; Chen et al., 2018; Mohsan Ullah Goraya et al., 2020).

### 9.2 Adaptive immune response

The adaptive immune response plays a critical role in controlling influenza B virus infection. The virus-specific antibodies and T cells are the main effectors of the adaptive immune response. The antibodies neutralize the virus by binding to the surface glycoproteins hemagglutinin and neuraminidase, preventing viral attachment to the host cells and promoting viral clearance. T cells recognize and eliminate virus-infected cells, preventing viral replication and spread (Houser, 2015).

### 9.3 Mechanisms of protection

Protection against influenza B virus infection is primarily mediated by virus-specific antibodies. The antibodies can protect via two mechanisms: neutralization and Fc-mediated effector functions. Neutralizing antibodies bind to the HA and NA surface glycoproteins, preventing viral attachment to the host cells and promoting viral clearance. Fc-mediated effector functions involve the binding of the Fc portion of the antibody to Fc receptors on immune cells, triggering the activation of these cells and promoting viral clearance (DiLillo et al., 2016).

### 9.4 Immune evasion

Influenza B viruses have developed several mechanisms to evade the host immune response. One of the primary mechanisms is antigenic drift, which involves the accumulation of mutations in the HA and NA surface glycoproteins, leading to the production of variant viruses that can evade the pre-existing immunity in the population. Another mechanism is antigenic shift, which involves the reassortment of gene segments between different influenza B viruses or between influenza A and B viruses, leading to the production of novel viruses that can cause pandemics (Walters et al., 2019).

**Figure 5:**
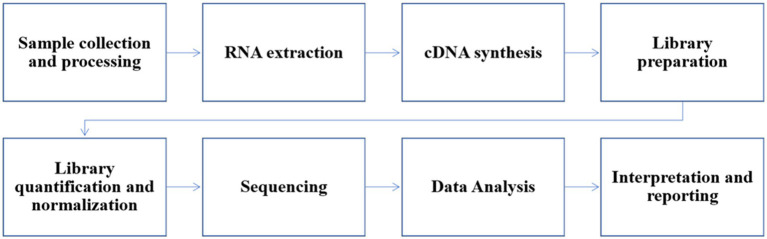
Workflow of NGS for influenza B samples.

The immune response to influenza B viruses is critical for controlling the infection and preventing severe disease. The innate and adaptive immune responses play complementary roles in the defense against the virus. Protection against influenza B virus infection is primarily mediated by virus-specific antibodies, which can protect neutralization and Fc-mediated effector functions. However, influenza B viruses have developed several mechanisms to evade the host immune response, including antigenic drift and shift. Further research is needed to develop more effective vaccines and antiviral therapies that can overcome these immune evasion mechanisms and provide long-lasting protection against influenza B viruses (Figure 5).

## 10 Antiviral strategies against IBV

There are two types of approved anti-influenza B drugs: neuraminidase inhibitors (NAIs) like zanamivir and oseltamivir, and adamantanes such as rimantadine and amantadine. Adamantanes inhibit the activity of the ion channel AM2, but not BM2. This is due to specific amino acid residues in the BM2 ion channel, which, being polar, decrease the drug’s access to the ion channel pore (Ma et al., 2008). As a result, adamantanes are ineffective against IBV. NAIs can inhibit IBV replication. The high degree of catalytic site conservation between IAV and IBV can be used to explain why NAIs have an inhibitory effect on both IAV and IBV. Burnham et al. (Burnham et al., 2014) have published a thorough study on the effectiveness of NAIs and the resistance mutations of IBVs. Oseltamivir is less effective in treating influenza B virus compared to its effectiveness against influenza A virus. On the other hand, zanamivir shows similar effectiveness against both influenza B virus and influenza A virus. Clinical isolates, surveillance isolates, and reverse genetics investigations all contain mutations that confer resistance to NAIs. Both the catalytic region of the enzyme (like R152K) and the framework residues (like D198N and I222T) might contain these changes. Contrary to the majority of resistance mutations, studies on the fitness cost of NAI-resistant mutations have demonstrated that the E119A and H274Y mutations have little effect on viral replication and fitness (Burnham et al., 2014). A new drug, baloxavir marboxil, has been developed against influenza A and B. This drug acts as a polymerase inhibitor that acts on influenza A and B. It’s interesting to note that distinct mutations in the NA gene from the two IBV lineages appear to have varied frequencies and effects (Farrukee et al., 2015).

## 11 Emerging vaccines

### 11.1 Vector base vaccines

These differences in influenza B virus infections can be explained by variations in how long immunity lasts after infection and how these viruses bind to specific sialic acid receptors in the respiratory tract. B/Victoria viruses have the ability to attach to both α-2,3- and α-2,6-linked sialic acids, whereas B/Yamagata viruses only attach to α-2,6-linked sialic acids. Research indicates that α-2,3-linked sialic acid glycans are more prevalent in the respiratory tissues of children than in adults. This disparity in receptor availability likely contributes to the observed age-specific patterns of infection between B/Victoria and B/Yamagata influenza viruses (Wang et al., 2012; Nicholls et al., 2007). Viral vectors represent a promising approach for vaccination due to their ability to efficiently deliver genetic material into cells at the injection site, facilitating the production of new antigens. This process triggers a robust immune response involving both humoral and cellular components, as the antigens are expressed at high levels in their native conformation. Additionally, viral vectors act as adjuvants by activating the innate immune system, thereby enhancing the adaptive immune response directed against the specific antigen (Ewer et al., 2016). Among viral vectors, those that are replication-deficient are considered the safest, although attenuated and fully replication-competent vectors are also utilized. Replicating vectors offer the advantage of increasing antigen expression through multiple replication cycles, although they must balance this benefit against potential immunogenicity from amplified vector antigens (Woolsey et al., 2023). Studies have shown that a single administration of a viral vector can induce durable immunity over time. One of the notable advantages of viral vector platforms over traditional seasonal influenza vaccines lies in their scalability for production. Viral vectors can be manufactured in large quantities using established cell lines in bioreactors, a capability that streamlines vaccine production. Notably, vaccines utilizing an attenuated yellow fever virus vector have already been developed and proven safe for human use (Chen and Wilson, 2020).

### 11.2 Nucleoside-modified mRNA-LNP vaccines

Nucleoside-modified mRNA-LNP vaccines targeting influenza B viruses (IBVs). Some studies explore the effectiveness of monovalent and pentavalent mRNA-LNP formulations encoding various IBV antigens, including hemagglutinin (HA), neuraminidase (NA), nucleoprotein (NP), and matrix protein 2 (M2). This includes the pentavalent vaccine’s ability to induce broadly protective immune responses against different IBV strains, outperforming individual antigen formulations (Arevalo et al., 2022). HA-specific antibodies contribute to cross-protection, NA-specific responses show lineage-specific efficacy. Additionally, NP vaccination elicits robust cellular immune responses, suggesting its potential role in protection against IBVs. Some recent studies underscore the promise of nucleoside-modified mRNA-LNP vaccines in combating influenza, particularly IBVs, and advocates for further investigation into multivalent mRNA-based vaccines for broader immune protection (Pardi et al., 2022).

### 11.3 mRNA vaccine

mRNA vaccines have undergone extensive refinement through experimental methods aimed at enhancing mRNA stability, delivery efficiency, and protein production. These advancements include the development of nanoparticle-based transport technologies that stabilize mRNA, improve cellular uptake, and enhance its biological availability upon entry into cells (Lee and Ryu, 2021). One significant advantage of mRNA vaccines is their ability to promote antigen expression without needing to enter the nucleus. This characteristic not only enhances their effectiveness in older populations but also significantly shortens the manufacturing timeline compared to traditional seasonal flu vaccines, making them highly attractive (Sayedahmed et al., 2020). Researchers have long been exploring mRNA platforms for influenza vaccines, demonstrating their immunogenicity and effectiveness against both closely matched and diverse virus strains. In non-human primate studies, lipid nanoparticle-delivered mRNA vaccines have elicited robust humoral responses comparable to those induced by licensed inactivated vaccines (Buschmann et al., 2021).

### 11.4 Live vaccine

Typically, to make traditional live influenza vaccines less potent, they are modified so that they can multiply at lower temperatures. The re-assortment of a cold-adapted virus with seasonal influenza A virus produces these cold-adapted virions. Virus attenuation may also be achieved by other means. Take the NS1-based escape mutants of the influenza virus as an example. By truncating or deleting the appropriate gene, they may be used to make live-attenuated versions of the virus. Alterations to the viral M1 protein also can enhance the immune system’s ability to defend mice against both related and unrelated influenza viruses (Thomas et al., 2009). The immune response induced by live attenuated vaccination is quite similar to the one seen in a real infection; it is both secretory (mucosal response) and systemic. Live AVs were formerly given to young children using nasal drops; however, this vaccine is now given intranasally. The immunogenicity of the two methods, however, is comparable. In children, the mucosal response to live attenuated vaccination is marked by the presence of IgA antibodies in nasal secretions. These antibodies reach their highest point 2 to 11 weeks after vaccination and gradually decline between 6 months and 1 year following immunization (Tennis et al., 2012).

## 12 Art Technologies

### 12.1 CRISPR

A rapid and sensitive method used for the identification of pathogens such as the influenza B virus and CRISPR technology is a tool in genetic engineering that allows the precise modification of DNA in organism (Rarey et al., 1984). It has a variety of application in the field of diagnostics. To identify IBV using CRISPR technology, specific RNA guides can be designed to target the genetic material of the virus. When the CRISPR system encounters the viral RNA or DNA, it can be programmed to cleave and disable the virus acting as a potential antiviral mechanism (Mukhopadhyay et al., 1992).

CRISPR-based diagnostic methods such as DETECTOR [DNA endonuclease-targeted CRISPR trans reporter] and SHERLOCK [specific high sensitivity enzymatic reporter UnLOCKing] systems have been developed for the identification of emerging viral infections. These CRISPR technologies are coupled with the reporter system to identify the specific nucleic acid sequence of viruses (Mukhopadhyay et al., 1992). Park et al. (2021) developed a CRISPR-cas12a-based assay for the identification of influenza A and B. 103 RNA copies of IBV or IAV could be detected from the CRISPR-cas12a-based assay. This method can detect the low titer values of IAV and IBV with high specificity and rapidly (Rarey, 1985). CRISPR technology can detect several respiratory pathogens like SARS-CoV 2, RSV, and influenza viruses with a sensitivity of around 90–100 and 90% specificity. The CRISPR technology could be used for the identification of influenza B virus and other respiratory viruses (Table 5).

**Table 5:** Comparison of influenza A and influenza B viruses.

Characteristics	Influenza A virus	Influenza B virus	References
Phylogenetics	Subtyped by NA and HA (e.g., H3N2 and H1N1)	According to HA, have two lineages: Yamagata and Victoria	Webster et al. (1992) and Arvia et al. (2014)
Host range	Likely animal hosts and many animal reservoirs	Animal reservoirs not established	Webster et al. (1992)
Age susceptibility	Most severe in elderly and children	Most severe in adolescence and children	Caini et al. (2019)
Epidemiology	Pandemic potential, global impact	Typically causes localized outbreaks, less global impact	Caini et al. (2019)
Clinical presentation	Range from mild to severe respiratory symptoms Can lead to complications such as pneumonia and bronchitis Avian strains caused severe diseases and high death rates	Similar symptoms to influenza A but milder Less likely to cause severe complications Caused severe health problems in children	Gaitonde et al. (2019); Banning (2005)
Genetic diversity	High genetic diversity, multiple subtypes and strains	Lower genetic diversity, primarily two lineages	Valesano et al. (2020)
Virology	13 known proteins and 8 segments	11 known proteins and 8 segments	Briedis and Lamb (1982), Hatta and Kawaoka (2003)
Immunology	M1 and NP are the major CD8+ T-cell antigens Generally, strong IFN-αβ and cytokine response HA is the major antibody target	Major CD8+ T-cell antigens are unknown strong IFN-αβ and cytokine response, details unclear HA is the major antibody target	Wu and Metcalf (2020)
Antivirals	Adamantanes used are effective NAIs effective, but resistance detected	Adamantanes are ineffective NAIs effective, but resistance detected, in some cases without apparent fitness cost	Scholtissek et al. (1998)

### 12.2 Next generation sequencing (NGS)

The history of next-generation sequencing (NGS) in the study of influenza B virus marks a significant evolution in virology. Influenza B virus, first isolated in 1940, was traditionally studied using culture-based techniques and Sanger sequencing (Sheng et al., 2018). However, the advent of NGS in the early 2000s, with technologies developed by companies like 454 Life Sciences and Illumina, transformed the field by enabling rapid, high-throughput sequencing of viral genomes (Kulski, 2016). The mid-2000s saw the first complete genomes of influenza B virus sequenced using NGS, providing a comprehensive understanding of its genetic diversity and evolution. This technological leap facilitated real-time genomic surveillance, allowing researchers to track the spread and mutations of the virus globally. Additionally, NGS enabled metagenomic studies, improving diagnostic capabilities by identifying influenza B virus in clinical samples without prior knowledge of its presence. During outbreaks, rapid sequencing data from NGS provided insights into transmission and outbreak sources. Further advancements in sequencing technologies, such as Oxford Nanopore and PacBio, have continued to enhance the resolution and accuracy of influenza B genome studies (Wang et al., 2021). NGS has also been crucial in optimizing influenza vaccines by providing detailed genetic information for selecting relevant strains. Global surveillance networks like the Global Initiative on Sharing All Influenza Data (GISAID) leverage NGS data to monitor virus evolution, improving preparedness for epidemics and pandemics. By integrating NGS with computational models, prediction into future influenza B virus trends have emerged, potentially leading to more effective vaccines and antiviral strategies. Shortly, NGS has profoundly impacted public health by enhancing our understanding of influenza B virus, improving diagnostic methods, and informing preventive measures.

## 13 Conclusion

Influenza B virus (IBV) stands as a significant pathogen in the history of respiratory illnesses, demanding continual research and vigilance to combat its impact on human health. The recent reported cases of influenza B highlight the decline in influenza B/Yamagata and the possibility of extinction. Therefore, it’s compulsory to review the new detection, prevention, and vaccine policies. This review highlights the various aspects of IBV, from its evolution and phylodynamic to diagnostic techniques, immunology, and antiviral strategies. IBV’s evolutionary history has been a fascinating topic of study because it has shown that it is capable of undergoing genetic alterations that can result in the formation of new strains and seasonal variations. Understanding the phylodynamic of the virus helps public health officials predict and prepare for potential outbreaks, as well as select the most suitable vaccine strains to include in seasonal influenza vaccines. Accurate and prompt diagnosis plays a crucial role in managing IBV cases effectively. The advent of advanced diagnostic techniques such as real-time PCR, RT-PCR, microassay, pyrosequencing, NASBA, LAMP, CRISPR and NGS has revolutionized the detection process, allowing for rapid identification of IBV strains and early intervention.

The immunology of IBV is a complex area of research, highlighting the interplay between the virus and the human immune system. Understanding the host immune response is critical to developing effective vaccines and antiviral therapies that can target IBV and reduce its impact on vulnerable populations. Moreover, antiviral strategies against IBV have seen significant advancements, with ongoing research into novel therapeutic approaches and the development of antiviral agents. Prophylactic measures, including annual influenza vaccination, play a pivotal role in mitigating the severity of IBV infections and preventing complications. Compared to influenza A, the greatest risk of influenza B to humans lies in its potential to cause severe illness in specific populations, such as children and the elderly, and its ability to co-circulate with influenza A, complicating prevention and treatment efforts. Despite the focus on influenza A due to its pandemic potential, studying influenza B is equally important as it contributes significantly to the annual flu burden, requires distinct vaccine considerations due to its two lineages, and poses unique challenges in public health, particularly in predicting and controlling seasonal outbreaks. As we conclude, it is clear that the influenza B virus remains a persistent public health concern worldwide. Collaborative efforts between virologists, immunologists, epidemiologists, and public health officials are essential in advancing our understanding of the virus and improving our preparedness to face future outbreaks. Continued research into IBV’s evolution, diagnostics, and immunology will empower us to stay one step ahead of the virus, safeguarding the well-being of global populations and reducing the burden on healthcare systems during influenza seasons.
